# Trophic Duality: Taxonomic Segregation and Convergence in Prey Functional Traits Driving the Coexistence of Apex Predators

**DOI:** 10.3390/biology15010031

**Published:** 2025-12-24

**Authors:** Hilton Entringer Jr, Ana Carolina Srbek-Araujo

**Affiliations:** 1 Centro para el Estudio de Sistemas Marinos (CESIMAR), Centro Nacional Patagónico (CENPAT), Consejo Nacional de Investigaciones Científicas y Técnicas (CONICET), Puerto Madryn 9120, Chubut, Argentina; 2 Facultad de Ciencias Naturales y Ciencias de la Salud, Universidad Nacional de la Patagonia San Juan Bosco (UNPSJB), Puerto Madryn 9120, Chubut, Argentina; 3 Laboratório de Ecologia e Conservação de Biodiversidade (LECBio), Programa de Pós-Graduação em Ciência Animal (PPGCA), Universidade Vila Velha (UVV), Vila Velha 29102-920, Espírito Santo, Brazil

**Keywords:** carnivore conservation, coexistence mechanisms, functional convergence, *Panthera onca*, *Puma concolor*, resource partitioning, trophic ecology

## Abstract

The coexistence of functionally similar predators offers a framework for understanding the mechanisms shaping ecological communities. Jaguars and pumas are broadly sympatric in the Neotropics, yet the processes sustaining their persistence remain unclear. Classical niche theory predicts that coexistence requires ecological differentiation, whereas modern models emphasize balancing stabilizing (reducing interspecific competition) and equalizing mechanisms (minimizing fitness differences). Although demographic components were not directly estimated, we integrated secondary dietary data compiled from 21 sympatric populations through a systematic literature review. This integration allowed us to identify trophic patterns consistent with coexistence mechanisms across taxonomic and functional prey axes. Analyses revealed strong taxonomic segregation at finer scales, with jaguars primarily consuming Artiodactyla, while pumas exploited a broader spectrum including Rodentia, Cingulata, and Pilosa. This divergence reduces direct competition, consistent with stabilizing resource partitioning. In contrast, high overlap in functional traits (e.g., body mass and locomotor habit) indicated functional convergence potentially increasing fitness equivalence, consistent with equalizing mechanisms. These dynamics suggested that jaguar–puma macroecological coexistence may be maintained by a dynamic balance between patterns consistent with stabilizing and equalizing mechanisms expressed within trophic niche axes. From a conservation perspective, strategies should move beyond species-specific approaches; preserving both taxonomic and functional prey diversity is essential to sustain the trophic requirements of predators and the ecosystems they regulate.

## Simple Summary

Jaguars and pumas are two large predators that live in the same areas across the Neotropics, yet how they manage to coexist remains unclear. Understanding their coexistence can help explain the processes that structure ecological communities. Classical ecological theory suggests that species coexist by utilizing different resources, while modern models highlight the balance between mechanisms that reduce direct competition and those that equalize differences between species. We analyzed dietary data from 21 populations where jaguars and pumas live together to investigate these mechanisms. We found differences in the types of prey consumed by each felid. Jaguars tend to primarily consume deer and tayassuids, whereas pumas eat a wider variety of prey, including large rodents, armadillos, and anteaters. This dietary segregation likely reduces direct competition between the two species. However, both predators share similar prey traits, such as body size and locomotor habit, which allows them to play similar ecological roles despite differences in specific prey. These findings are consistent with the idea that jaguar–puma coexistence may be supported by a combination of resource partitioning and functional similarity. From a conservation perspective, protecting the diversity of prey species and their ecological interactions is likely to be essential to support both predators and may help maintain the health and balance of Neotropical ecosystems.

## 1. Introduction

A fundamental challenge in ecology is to understand how functionally similar species coexist, given that their similarity makes them particularly susceptible to competitive exclusion [1,2]. This, the competitive exclusion principle posits that shared reliance on limited resources will inevitably lead to the displacement of one species unless they differentiate their ecological niches [3,4]. The ecological niche, defined as the set of all environmental requirements for a species’ persistence [5], offers multiple dimensions for differentiation, including space, time, and trophic resources [4]. Among these, trophic partitioning is especially important because it directly reduces the energetic and behavioral costs of competition [6,7]. By segregating their diets, competing species reduce conflict and exploit resources more efficiently, establishing a stable foundation for persistence in shared landscapes [2,3,6].

Contemporary coexistence models highlight that the persistence of potentially competing species depends on two main groups of complementary mechanisms: stabilizing and equalizing mechanisms. Stabilizing mechanisms promote coexistence through niche partitioning, which grants each species a competitive advantage over specific resources [2,8]. This process intensifies intraspecific regulation relative to interspecific effects, which reduce competition [2]. Equalizing mechanisms, in turn, diminish absolute fitness differences and make species more similar in competitive ability, slowing exclusion [2,8]. In operational terms, stabilizing mechanisms reduce niche overlap, whereas equalizing mechanisms reduce competitive inequalities. Because niche dimensions are interdependent [4,5], stabilizing and equalizing mechanisms expressed in one dimension (e.g., space and time) may reverberate in others, including diet [2]. Because the trophic niche is itself multidimensional, this same logic of trade-offs can apply internally among its components, where a partitioning along one axis can act as a stabilizing force that, in turn, permits significant overlap along another as an equalizing effect. Overall, this conceptual structure highlights that dietary divergence and convergence should be interpreted as complementary outcomes of interacting coexistence mechanisms rather than mutually exclusive patterns.

Apex predators exert a disproportionate influence on the structure and functioning of ecological communities, acting as trophic engineers that regulate both direct and indirect interactions within food webs [9,10,11]. Through direct predation and cascading effects, they influence not only the abundance and composition of their prey but also the dynamics of lower trophic levels [9,10,11]. Because many apex predators share convergent morphological traits and comparable energetic requirements [12,13], the potential for intense competition within their guilds is exceptionally high [14]. In such contexts, dominant predators can suppress subordinate ones through intraguild predation, interference, or behavioral exclusion, processes that structure access to shared resources [14,15,16,17]. These pressures often induce adaptive adjustments by subordinate species, including temporal or spatial shifts and trophic niche differentiation, which mitigate direct conflict and enable coexistence [14,18]. Apex predators, therefore, integrate top-down regulation with strong interspecific interactions, often modulating trophic niches through adjustments in other ecological dimensions. Consequently, these guilds constitute an ideal framework for investigating how stabilizing and equalizing mechanisms sustain coexistence.

In the Neotropics, jaguars (*Panthera onca*) and pumas (*Puma concolor*) are sympatric from Mexico to northern Argentina, though pumas range more widely, from Canada to southern Chile [19,20]. Their ranges broadly overlap, with the jaguar’s distribution largely nested within the puma’s broader range. Both species have undergone range contractions and population declines driven by habitat loss, fragmentation, roadkill, and human conflict. The jaguar is particularly affected, having disappeared from about half of its historical range [20]. The puma, though extirpated in some regions, still occupies most of its original range [19].

Jaguars weigh on average about 80 kg, with some individuals exceeding 130 kg in the Pantanal [21], whereas pumas average around 60 kg [22] and can reach approximately 100 kg at higher latitudes [23]. This size disparity may establishes a competitive hierarchy, with the larger carnivore typically occupying the dominant role in sympatric contexts [15,24]. Recent continent-wide analyses show that pumas increase their reliance on large prey in regions where they function as the dominant predator, but this tendency is suppressed where jaguars are present, reflecting the constraining influence of hierarchical dominance between this pair of predators [25]. While these macroecological analyses indicate broad dietary constraints, the fine-scale trophic dynamics within coexistence zones remain poorly resolved. An integrative assessment of partitioning across trophic niche axes within coexistence zones is therefore essential for identifying the mechanisms that sustain coexistence. Such an approach may clarify how variation along one axis can shape patterns along another and helps connect local trophic strategies with broader macroecological trends.

Herein, we integrated dietary data from the literature to characterize the trophic strategies of jaguars and pumas across their sympatric range. We hypothesized that jaguar dominance would drive stabilizing niche differences via taxonomic partitioning, while their shared role as large felids would maintain competitive equivalence through functional convergence in prey traits. Specifically, we expected (i) lower taxonomic overlap and stronger centroid separation at finer prey levels and (ii) consistently high overlap in prey functional traits across coexistence scenarios. Based on populations sampled simultaneously within the same areas, we (i) quantified the scale-dependent (across prey taxonomic and functional traits) patterns of niche overlap, breadth, and their interactions to assess the balance between dietary patterns consistent with equalizing (convergence) and stabilizing (divergence) mechanisms, and (ii) analyzed the compositional structure of the diet and its population-level variability to identify the specific prey axes and compensatory mechanisms that enable this coexistence. Because our analyses rely on diet composition rather than demographic data, we treat stabilizing and equalizing mechanisms in a heuristic sense rather than attempt a formal decomposition of coexistence in the strict Chesson (2000) framework [2]. Our integrated approach therefore provides an empirical trophic context for evaluating patterns consistent with these two classes of mechanisms at macroecological scales. Additionally, we discuss the implications of these interactions for the conservation of both large felids.

## 2. Materials and Methods

### 2.1. Data Collection

**Figure 1:**
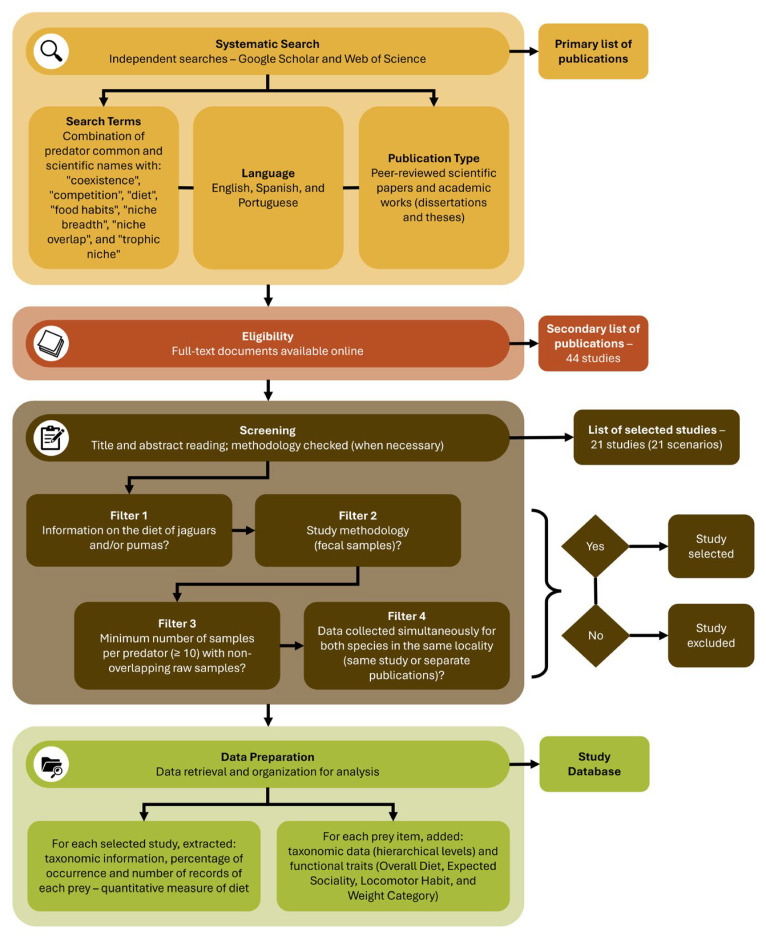
Methodology for the systematic literature search and selection criteria used in the present study to identify studies on the trophic ecology and diet composition of jaguars and pumas across their geographic range.

We conducted a systematic literature search to identify studies reporting the trophic ecology and diet composition of jaguars and pumas (Figure 1). We independently performed searches in July 2025 in Google Scholar and Web of Science, using combinations of predators common and scientific names and ecological terms such as “coexistence”, “competition”, “diet”, “food habits”, “niche breadth”, “niche overlap”, and “trophic niche”. We considered publications written in English, Spanish, and Portuguese, ensuring coverage of the most widely spoken languages across the predators’ distribution. Peer-reviewed scientific papers were prioritized, but academic works (dissertations and theses) were also included to increase geographic replication. No temporal restrictions on publication year were applied, allowing the inclusion of all relevant studies published up to the time of the search.

We considered only full-text documents available online that provided dietary information for both jaguars and pumas collected simultaneously within the same locality, even when data originated from separate publications. We applied these criteria to ensure spatial and temporal coupling of diets within sites, allowing robust comparisons of trophic strategies in coexistence contexts (here referred to as scenarios). For this, we included studies based on fecal samples to ensure methodological consistense among scenarios, and with a minimum sample size of 10 scats per predator. We excluded studies relying solely on other methods (e.g., stomach contents, kills, direct observations), and studies employing mixed methods were only if fecal-based data could be distinguished and extracted separately. To avoid pseudoreplication, when more than one study used the same raw samples, we retained only the most relevant publication (scientific paper and/or study with the highest number of samples). Because the studies varied in sampling effort and sampling period or seasonality, we did not standardize these aspects to include as many relevant studies as possible. Similarly, we did not restrict studies to particular habitat types or environmental conditions to maximize geographic coverage across the predators’ distribution ranges.

**Figure 2:**
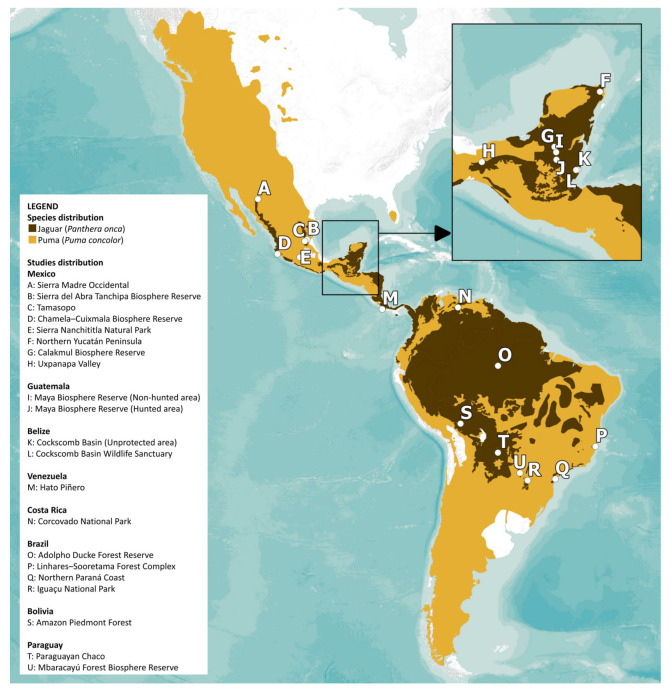
Current geographic distribution of jaguar (*Panthera onca*) and puma (*Puma concolor*), showing the location of the 21 coexistence scenarios analyzed in the present study (Mexico: Sierra Madre Occidental [26], Sierra del Abra Tanchipa Biosphere Reserve [27], Tamasopo [28], Chamela–Cuixmala Biosphere Reserve [29], Sierra Nanchititla Natural Park [30,31], Northern Yucatán Peninsula [32], Calakmul Biosphere Reserve [33], Uxpanapa Valley [34]; Guatemala: Maya Biosphere Reserve (Non-hunted area) and Maya Biosphere Reserve (Hunted area) [35]; Belize: Cockscomb Basin (Unprotected area) and Cockscomb Basin Wildlife Sanctuary [36]; Venezuela: Hato Piñero [37]; Costa Rica: Corcovado National Park [38]; Brazil: Adolpho Ducke Forest Reserve [39], Linhares–Sooretama Forest Complex [40,41], Northern Paraná Coast [42], Iguaçu National Park [43]; Bolivia: Amazon Piedmont Forest [44]; Paraguay: Paraguayan Chaco [45], Mbaracayú Forest Biosphere Reserve [46]). The studies used and the diet details they provided are also listed in Tables S1 and S2.

We included 21 studies that fulfilled our selection criteria in the analysis (two pairs of studies addressed one distinct scenario each, two involved two different scenarios each, and 15 addressed a single scenario distinct from the previous ones; Figure 2; Table S1), selected from 44 eligible studies. From each source, we extracted taxonomic information, the percentage of occurrence and the number of records of each prey as a quantitative measure of diet (Table S2). The number of records was specifically retrieved to allow for the subsequent recalculation of occurrence percentages when merging results from the same scenario across studies.

### 2.2. Data Analysis

#### 2.2.1. Prey Classification and Data Treatment

We conducted the analyses separately for two complementary prey classification schemes, one taxonomic and the other functional. The taxonomic classification included four hierarchical levels (Class, Order, Family, and Genus) (Table S2), representing increasing ecological resolution. This structure allowed us to evaluate patterns at each hierarchical level and identify the scales at which divergence emerges. We also classified consumed taxa according to functional traits, considering Overall Diet (carnivore, folivore, frugivore, granivore, insectivore, omnivore, and their combinations), Expected Sociality (gregarious, in pairs/small groups, solitary/gregarious, solitary/pairs, and solitary), Locomotor Habit (aquatic, arboreal, scansorial, semiaquatic, terrestrial, terrestrial/fossorial, and volant), and Weight Categories (categorizing each taxon based on mean body mass into: <1.0 kg, 1.0–4.9 kg, 5.0–14.9 kg, 15.0–24.9 kg, 25.0–34.9 kg, 35.0–44.9 kg, 45.0–54.9 kg, 75.0–84.9 kg, 85.0–94.9 kg, and >95.0 kg) (Table S2). For taxonomic standardization and functional trait information, we first relied on data from the GBIF (Global Biodiversity Information Facility) [47], followed by the IUCN (International Union for Conservation of Nature database) [48]. When information was not available in these global databases, we consulted specialized literature to complement the dataset.

Based on the cited classifications, we aggregated prey consumption values (percentage of occurrence) from the data sources for each category and for each predator population (Table S2). We excluded occasional records of plants and invertebrates, as these items are not part of the typical diet of large felids and provide negligible energetic contribution [13]. Subsequently, we rescaled the percentage of occurrence of the remaining prey items to sum to 100%.

We described all statistical analyses below according to their specific application, using the percentage of occurrence values as the analytical basis.

#### 2.2.2. Statistical Analyses

Our analytical approach was designed to frame dietary patterns considering Chesson’s (2000) [2] coexistence theory, connecting trophic variation across taxonomic scales and functional categories to indices that we interpret as being more consistent with stabilizing or equalizing mechanisms. We first quantified mean interspecific niche overlap as the primary indicator of equalizing (high values) versus stabilizing (low values) patterns through the prey-defined categories. We quantified interspecific niche overlap for each scenario and prey classification using Pianka’s index [49], with values ranging from 0 (no overlap) to 1 (total overlap).

We then estimated trophic niche breadth separately for each prey classification and for each predator population using Levin’s index standardized by Hurlbert’s formula [50], which ranges from 0 (maximum specialization) to 1 (maximum generalization). To examine whether the two felids exhibited coordinated or independent niche breadth adjustments, we fitted beta regression models, rescaling niche metrics to the open (0, 1) interval to satisfy beta regression assumptions [51]. For this, we used a reciprocal approach, modeling puma niche breadth as the response variable and jaguar as the predictor, and vice versa. This bidirectional formulation captures the degree of niche breadth concordance between predators while explicitly identifying the effect size of the species treated as the predictor. For each model, we extracted 95% confidence intervals directly from the estimated lower and upper bounds of the coefficient, providing an explicit quantification of effect-size uncertainty. For families of related models (e.g., across taxonomic levels or functional dimensions), we adjusted *p*-values using the Benjamini–Hochberg false-discovery rate (FDR) procedure to control for multiple testing [52]. We interpreted the results based on the nature of the association: (i) a significant positive relationship was interpreted as a pattern consistent with equalizing mechanisms, where both predators expand or contract their niches in parallel; (ii) a neutral association indicates stabilizing coexistence through independent adjustments; and (iii) a significant negative relationship was interpreted as a pattern consistent with strong stabilizing mechanisms, where the predators make divergent adjustments in their niche breadth.

Subsequently, we evaluated the influence of niche breadth on the degree of niche overlap to determine how variation in trophic specialization translates into effective resource partitioning. We fitted separate beta regression models for each predator (applying the same rescaling, confidence interval extraction, and FDR correction described earlier), testing how its niche breadth predicted the extent of dietary overlap. This analysis is grounded in classic niche theory, which posits that specialization levels regulate the intensity of interspecific competition [3]. We classified the resulting relationships based on their effect: (i) a negative relationship was interpreted as being consistent with a stabilizing mechanism, where niche expansion reduces resource sharing; (ii) a neutral association was interpreted as overlap independent of niche breadth, reflecting a balance of equivalence; (iii) a symmetrical positive relationship was interpreted as a pattern consistent with equalizing mechanisms, where generalization predictably increases shared prey use; and (iv) an asymmetrical positive effect was interpreted as a destabilizing dynamic, in which one predator’s niche expansion increases overlap to the detriment of the other.

To complement the univariate metrics of niche breadth and overlap, we employed multivariate analyses to characterize the compositional mechanisms and compensatory variability shaping the observed macroecological patterns. We assessed diet composition using Non-metric Multidimensional Scaling (NMDS) based on Bray–Curtis dissimilarity matrices. We evaluated the goodness-of-fit of the NMDS by its stress value, where values below 0.2 indicate a reliable ordination [53]. First, to test for significant differences in diet composition (centroid separation) between predator populations for each prey classification, we applied a Permutational Multivariate Analysis of Variance (PERMANOVA) [54] with 999 permutations. To identify the prey categories most strongly structuring the dietary patterns, we fitted vectors onto the NMDS ordinations using the envfit function when reaching a significance of *p* ≤ 0.05. Second, we evaluated the homogeneity of multivariate dispersions using the Permutational Analysis of Multivariate Dispersion (PERMDISP) [54] with 999 permutations. We used this as a measure of beta diversity, indicating the degree of dietary plasticity (flexibility) among each predator’s set of populations. For both PERMANOVA and PERMDISP, we also applied the Benjamini–Hochberg FDR procedure. We used the joint interpretation of PERMANOVA and PERMDISP results to classify dietary patterns as more consistent with particular coexistence mechanisms: (i) Centroid separation with symmetrical dispersion was interpreted as consistent with robust stabilizing differences reinforced by similar species-level constraints; (ii) Centroid separation with asymmetrical dispersion suggests stabilizing differences combined with a compensatory equalizing mechanism (asymmetrical flexibility); (iii) Centroid overlap with symmetrical dispersion was consistent with a pure equalizing mechanism, in which predators are functionally similar and maintain comparable levels of population flexibility; and (iv) Centroid overlap with asymmetrical dispersion suggests a pattern consistent with equalizing mechanisms maintained by compensatory flexibility.

We performed analyses in R (version 4.4.1) [55], adopting a significance level of α = 0.05 for all tests. We implemented NMDS ordinations, PERMANOVA, envfit vectors, and PERMDISP using the vegan package [56], whereas we fitted beta regression models with the betareg package [50].

## 3. Results

### 3.1. Taxonomic Patterns of Prey

**Figure 3:**
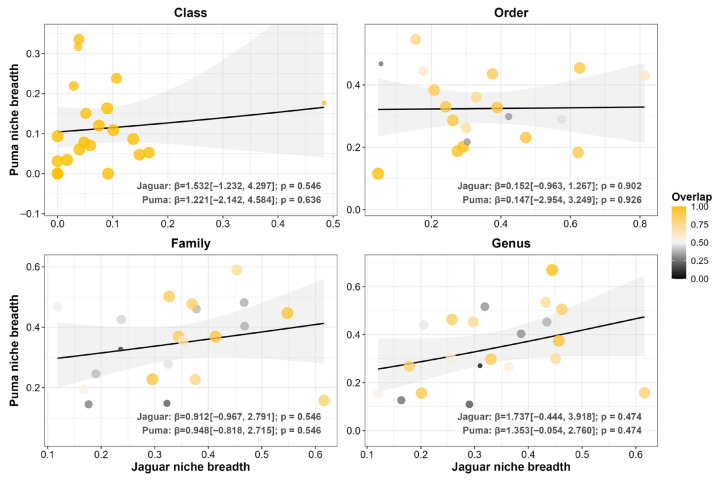
Beta regression models exploring the relationship between jaguar (*Panthera onca*) and puma (*Puma concolor*) taxonomic niche breadth. The analysis was performed across 21 coexistence scenarios. Each panel represents a different taxonomic level of prey classification (Class, Order, Family, and Genus). Each point represents a single coexistence scenario, with its size and color indicating the degree of niche overlap. Lines represent the predictive relationship from the GLM beta, and the shaded area indicates the 95% confidence interval. Beta coefficients (β) and *p*-values are shown in each panel.

Across the 21 coexistence scenarios (Figure 2), taxonomic niche overlap followed a clear scale-dependent pattern. Consistent with our first prediction, taxonomic divergence increased at finer prey levels. At the Class level, we found that overlap was nearly complete (0.993 ± 0.011) and niche breadth was lowest (jaguar = 0.082 ± 0.105 vs. puma = 0.113 ± 0.098), suggesting a broad-scale equalizing pattern driven by shared specialization on the same classes. This high overlap declined at finer resolutions (Order: 0.742 ± 0.239; Family: 0.589 ± 0.261; Genus: 0.609 ± 0.278), marking a shift toward an emergent stabilizing pattern. Parallel to this, we observed that niche breadths expanded relative to the Class level but remained narrow overall (Order: jaguar = 0.352 ± 0.193 vs. puma = 0.324 ± 0.113; Family: 0.342 ± 0.128 vs. 0.348 ± 0.132; Genus: 0.332 ± 0.124 vs. 0.344 ± 0.155), patterns that align with a stabilizing mechanism in which populations of each species tend, on average, to specialize on distinct prey. Moreover, we found no significant interspecific concordance in specialization (*p* ≥ 0.474; Figure 3), which is consistent with stabilizing patterns through independent adjustments in niche breadth.

**Figure 4:**
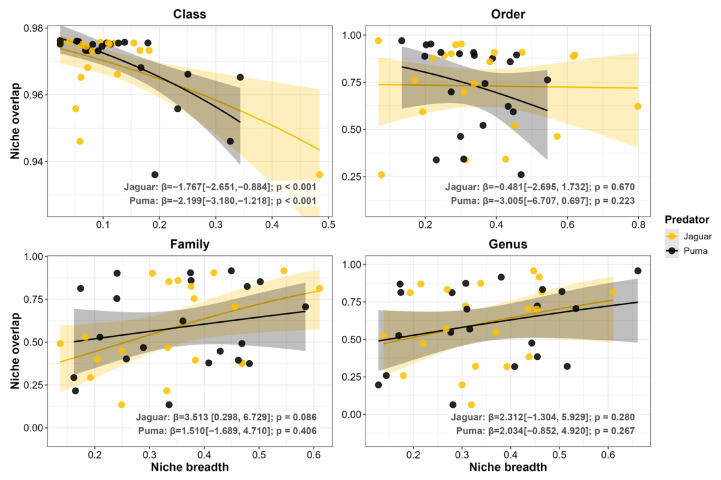
Beta regression models exploring the relationship between niche overlap and niche breadth for both jaguar (*Panthera onca*) and puma (*Puma concolor*). The analysis was performed across 21 coexistence scenarios. Each panel represents a different taxonomic level of prey classification (Class, Order, Family, and Genus). Each point represents a predator population in a single coexistence scenario. Lines represent the predictive relationship from the GLM beta for each species, and the shaded area indicates the 95% confidence interval. Beta coefficients (β) and *p*-values are shown in each panel.

When we examined the effect of specialization on overlap, we observed a symmetrical stabilizing pattern at the Class level, where broader diets significantly reduced interspecific overall overlap for both predators (*p* < 0.001 for both; Figure 4). Despite this broad-scale stabilizing pattern, we detected neutral effects at all finer scales for both jaguars and pumas (*p* ≥ 0.086; Figure 4).

**Figure 5:**
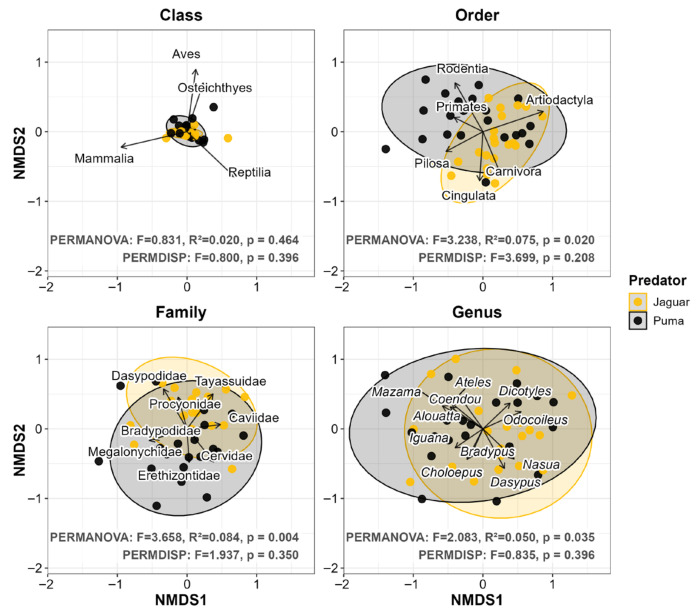
Non-metric Multidimensional Scaling (NMDS) of diet composition for jaguar (*Panthera onca*) and puma (*Puma concolor*). The analysis was performed across 21 coexistence scenarios. Each panel represents a different taxonomic level of prey classification (Class, Order, Family, and Genus). Each point corresponds to a single predator population in a coexistence scenario, while ellipses depict the multivariate dispersion of samples for each predator. PERMANOVA and PERMDISP results are presented in each panel. Vectors identified influential prey items in the ordination (*p* ≤ 0.05); however, to ensure graphical clarity, only vectors with r^2^ ≥ 0.200 were plotted). Ordination stress values indicated an accurate representation of dietary dissimilarities for Class (0.045) and Order (0.179), and an acceptable resolution for Family (0.239) and Genus (0.200).

When we analyzed prey composition, our results reinforced the previously observed patterns. At the Class level, we observed a similar overall interspecific consumption pattern (PERMANOVA: *p* = 0.464; Figure 5). However, within this broad dietary similarity, we detected significant centroid separations at the Order, Family and Genus levels (PERMANOVA: *p* ≤ 0.035), revealing clear trophic divergence at finer resolutions. We found that mammals were the most consumed prey by both predators, but jaguar diets were mainly associated with Artiodactyla (r^2^ = 0.836) and Carnivora (r^2^ = 0.483), whereas pumas were associated with Rodentia (r^2^ = 0.653), Cingulata (r^2^ = 0.499), Pilosa (r^2^ = 0.361), and Primates (r^2^ = 0.213; all *p* ≤ 0.011). Families and genera within these orders were the main contributors to the trophic differentiation (Figure 5). Although we observed that pumas tended to exhibit greater dietary dispersion than jaguars in all taxonomic scales (Class: jaguar = 0.060 vs. puma = 0.078; Order: 0.320 vs. 0.392; Family: 0.421 vs. 0.478; Genus: 0.511 vs. 0.545), we found no statistical differences in dispersion (PERMDISP: *p* ≥ 0.208; Figure 5). The combination of this fine-scale centroid separation and this symmetrical population-level dispersion suggests a stabilizing pattern in which divergent resource use was reinforced by similar species-level constraints.

### 3.2. Prey Functional Traits

**Figure 6:**
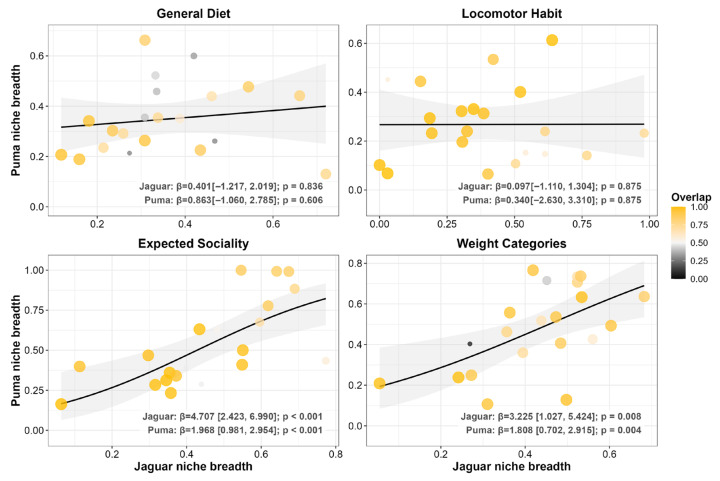
Beta regression models exploring the relationship between puma (*Puma concolor*) and jaguar (*Panthera onca*) functional niche breadth. The analysis was performed across 21 coexistence scenarios. Each panel represents a different functional category of prey (general diet, locomotor habit, expected sociality, and weight categories). Each point represents a single coexistence scenario, with its size and color indicating the degree of niche overlap. Lines represent the predictive relationship from the GLM beta, and the shaded area indicates the 95% confidence interval. Beta coefficients (β) and *p*-values are shown in each panel.

Consistent with our second prediction, functional overlap remained high across all prey trait categories. Contrary to the taxonomic axis, we found that the trophic dimension related to prey functional traits was dominated by equalizing patterns. This was evident in the mean niche overlap, which remained consistently high across all functional traits (Overall Diet: 0.685 ± 0.232; Locomotor Habit: 0.879 ± 0.156; Expected Sociality: 0.848 ± 0.186; and Weight Categories: 0.801 ± 0.223). Positive interspecific concordance in specialization levels for Expected Sociality and Weight Categories reinforced this context. While we found these associations to be significant in both model directions (*p* ≤ 0.008 for both predators), indicating mutually synchronized niche adjustments, albeit of unequal magnitude. The association was substantially stronger when jaguar niche breadth predicted puma breadth (Expected Sociality: β = 4.707; Weight Categories: β = 3.225) compared to the reciprocal effect of pumas on jaguars (Expected Sociality: β = 1.968; Weight Categories: β = 1.808). For the remaining traits, we observed neutral associations (Locomotor Habit and Overall Diet, *p* ≥ 0.606; Figure 6), consistent with secondary stabilizing pattern maintained by independent adjustments between predators.

**Figure 7:**
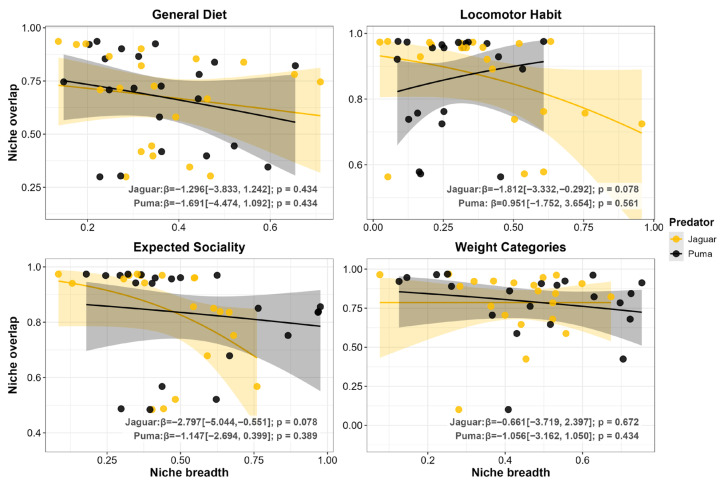
Beta regression models exploring the relationship between niche overlap and niche breadth for both jaguars (*Panthera onca*) and pumas (*Puma concolor*). The analysis was performed across 21 coexistence contexts. Each panel represents a different functional category of prey (general diet, locomotor habit, expected sociality, and weight categories). Each point represents a predator population in a single coexistence context. Lines represent the predictive relationship from the GLM beta for each species, and the shaded area indicates the 95% confidence interval. Beta coefficients (β) and *p*-values are shown in each panel.

When we examined the effect of niche breadth on overlap, we observed neutral relationships for all functional traits and both predators (*p* ≥ 0.078; Figure 7), suggesting a condition of equivalence where the level of niche overlap is independent of variations in the species’ trophic breadth.

**Figure 8:**
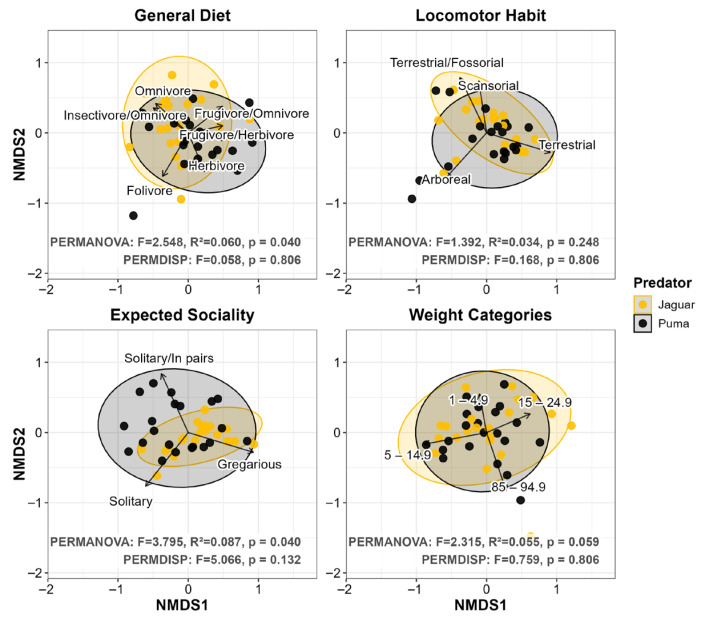
Non-metric Multidimensional Scaling (NMDS) of diet composition for jaguar (*Panthera onca*) and puma (*Puma concolor*). The analysis was performed across 21 coexistence scenarios. Each panel represents a different functional category of prey (general diet, locomotor habit, expected sociality, and weight categories). Each point corresponds to a single predator population in a coexistence scenario, while ellipses depict the multivariate dispersion of samples for each predator. PERMANOVA and PERMDISP results are presented in each panel. Vectors identified influential prey categories in the ordination (*p* ≤ 0.05); however, to ensure graphical clarity, only vectors with r^2^ ≥ 0.200 were plotted). Ordination stress values indicated an accurate representation of dietary dissimilarities for Locomotor Habit (stress = 0.080), Expected Sociality (stress = 0.080), and Weight Categories (stress = 0.180), and an acceptable resolution for General Diet (stress = 0.213).

Despite the broad equivalence evidenced by the univariate metrics, our multivariate analysis of functional diet composition revealed further secondary coexistence mechanisms operating within this apparent equalizing context. For Locomotor Habit and Weight Categories, we observed no centroid separation (*p* ≥ 0.059) and we found that dispersion remained symmetrical (mean dispersion: jaguar = 0.246 vs. puma = 0.267; *p* = 0.806, jaguar = 0.364 vs. puma = 0.326; *p* = 0.806, respectively; Figure 8). These patterns were consistent with a pure equalizing mechanism. Conversely, we detected significant centroid separation for General Diet and Expected Sociality (*p* = 0.040 for both) combined with symmetrical dispersion (General Diet: jaguar = 0.369 vs. puma = 0.378; *p* = 0.806; Expected Sociality: jaguar = 0.204 vs. puma = 0.283; *p* = 0.132; Figure 8), suggesting a pattern consistent with a robust stabilizing mechanism where the differentiated use of functional groups reduces overlap without increasing intraspecific variability. For General Diet, folivore (r^2^ = 0.515), omnivore (r^2^ = 0.391), and insectivore/omnivore (r^2^ = 0.372) categories contributed most to the jaguar grouping, whereas frugivore/omnivore (r^2^ = 0.382), herbivore (r^2^ = 0.355) and frugivore/herbivore (r^2^ = 0.253) categories contributed most to the puma grouping (Figure 8). For Expected Sociality, gregarious prey (r^2^ = 0.944) contributed most to the separation toward jaguars, while the solitary (r^2^ = 0.951) and solitary/pair (r^2^ = 0.853) categories contributed to the differentiation toward pumas (Figure 8). All cited vectors were significant (*p* ≤ 0.005).

## 4. Discussion

The coexistence of jaguars and pumas at the macroecological scale appears to be associated with a dynamic balance between trophic patterns that we interpreted as being consistent with stabilizing and equalizing mechanisms. Key stabilizing patterns were observed for the taxonomic axis, where predators diverged in dietary composition at fine taxonomic scales, although a strong convergence occurred in the consumption of higher-order taxonomic groups. The functional axis was dominated by equalizing patterns, resulting from the high similarity in prey functional traits, while a set of secondary mechanisms both reinforced these equalizing patterns and shifted toward stabilizing ones. This reveals that segregation and convergence may operate in parallel and alternately across trophic axes, forming a multiscale mechanism that may help sustain both the persistence and the ecological roles of these large felids, in a way that is qualitatively consistent with modern coexistence theory [2], even though we do not estimate stabilizing and equalizing components in the strict demographic sense. Under this perspective, the heterogeneity of strategies across the distribution of both predators (reflected here in the variation of patterns observed among different geographic and ecological contexts) may be one of the factors enabling the coexistence of jaguars and pumas at broader spatial scales.

### 4.1. Taxonomic Segregation: A Predominantly Stabilizing Force in Coexistence

Jaguar and puma coexistence relies on a clear segregation in their diets when evaluated taxonomic composition, a pattern that becomes statistically robust at fine taxonomic resolutions. While both predators concentrate their diets on mammals, which increases niche overlap at the Class level, jaguars specialize more strongly on Artiodactyla. In turn, pumas consume a wider range of orders in greater frequency than jaguars, particularly Rodentia, Pilosa, and Cingulata. This pattern in fine taxonomic levels may reduce direct competition, reflecting a central principle of community ecology and a main mechanism allowing the coexistence of ecologically similar species [2,3,6]. Analogous dynamics are observed in other systems. In African savannas of Kruger (South Africa) and Limpopo (Mozambique) National Parks, lions (*Panthera leo*) concentrate on large ungulates such as buffalo (*Syncerus caffer*), whereas the subordinate leopard (*Panthera pardus*) exploits a broader diversity of smaller prey, although in both areas herbivorous mammals remain the primary dietary components for both predators [57]. Similarly, in Nepal Chitwan National Park, dominant tigers (*Panthera tigris*) focus on mammals, primarily from the order Artiodactyla (such as chital, *Axis axis*, sambar deer, *Rusa unicolor*, and wild pig, *Sus scrofa*), while subordinate leopards mitigate competition by shifting to smaller Artiodactyla (muntjac, *Muntiacus* sp., and domestic goats, *Capra hircus*) and Primates (langurs, *Semnopithecus* sp., and rhesus monkeys, *Macaca mulatta*) [58]. Such examples suggest that large felids, including jaguars and pumas, persist through refined stabilizing mechanisms (prey partitioning), even while remaining anchored to an equalizing process at broad taxonomic groups across different coexistence scenarios.

Our analysis of taxonomic variability indicates a potential asymmetry in trophic plasticity. Pumas exhibited a broader prey base (87 species and 65 genera vs. 69 and 58 for jaguars; Table S2) and consistently greater dietary dispersion. Although this latter trend was not statistically significant, the overall pattern of greater flexibility strongly aligns with the puma established ecological profile as a highly adaptable predator capable of shifting diets across its extensive range [59]. This inherent versatility may represent a key adaptive strategy for persistence under competitive subordination [25,58,60], driving taxonomic segregation by allowing pumas to exploit prey less targeted by jaguars. In Yellowstone, for instance, pumas display high dietary flexibility to reduce direct competition and to facilitate coexistence with dominant wolves (*Canis lupus*) and bears (*Ursus* spp.) [60]. This strategy is also evident in other systems, where subordinate species such as the cheetah (*Acinonyx jubatus*) and the African wild dog (*Lycaon pictus*) adjust their foraging strategies to reduce overlap with larger predators, such as lions and leopards [61]. In this sense, trophic plasticity emerges as a critical mechanism for sustaining coexistence in competitive contexts [2,6]. Therefore, the puma greater potential flexibility could function as this counterbalance, facilitating its persistence under a subordinate position.

Moreover, the competitive dynamics mediated by variations in specialization and trophic overlap are strongly scale-dependent. At broader levels, such as Class, diversified diets in both species reduce overlap, suggesting that a simultaneous expansion of the dietary spectrum decreases direct competition by targeting different taxonomic groups. This pattern is consistent with classical niche theory [6]. However, we propose that this differentiation at the Class level may alleviate the competitive pressure at finer resolutions, leading to the observed neutral effects for both predators on overlap. Therefore, once major taxonomic distinctions are established, populations of each species tend to specialize on distinct prey independently, without necessitating further synchronized adjustments to minimize overlap at the macroecological scale. This strategy of independent adjustment is also confirmed by the lack of interspecific concordance in specialization levels. These results indicate that coexistence at the geographic scale emerges from a hierarchical filtering process where the establishment of niche partitioning at the Class level reduces the imperative for strict constraints at finer scales, resulting in a pattern of independent specialization strategies.

Thus, resource partitioning between jaguars and pumas at fine taxonomic scales, combined with adaptive strategies that neutralize competitive asymmetries, allows coexistence to occur even under marked overlap at broader levels. Segregation in prey identity, in particular, represents a process essentially related to a stabilizing mechanism [2] and reinforces the classical principle of limiting similarity [3]. Ultimately, the interaction between classical niche constraints and the stabilizing interpretation of our taxonomic results suggests a trophic system in which divergence in prey use, structured by spatial heterogeneity, may help sustain coexistence at the macroecological scale.

### 4.2. Functional Convergence: Patterns Consistent with an Equalizing Effect on Coexistence

Contrarily to the segregation observed along the taxonomic axis, the functional prey groups analysis revealed a consistently high niche overlap between jaguars and pumas. This pattern suggests that the intense overlap of functional prey groups is facilitated by the differentiated consumption of specific taxa. This may create a competitive refuge allowing for convergence on broader functional traits, a principle consistent with classical niche theory [6].

However, although the observed overlap may reflect the functional similarity of the two apex predators studied, this high overlap cannot not be interpreted as a simple functional redundancy of the prey consumed. As warned by Rosenfeld (2002) [62], true functional differentiation is often hidden in an n-dimensional niche space, and apparent redundancy usually disappears when multiple axes are evaluated. Our multivariate analyses confirm this. Even within this high functional sharing, the competitive hierarchy continues to structure the interaction, manifesting as a mosaic of secondary mechanisms. We found a robust stabilizing divergence in Overall Diet and Expected Sociality. Consistent with greater energetic demands in larger predators [13], the jaguars’ tendency to focus on both gregarious prey and on omnivore and herbivore functional groups may be related to energetic advantages. This pattern is analogous to that observed in lions in the Serengeti, which preferentially hunt certain gregarious species, a pattern explained by encounter-rate driven profitability in optimal diet models [63]. This reliance on high-profitability prey creates crucial niche refugia for the puma along these same trait (e.g., solitary and in-pair prey, as well as folivore and frugivore/herbivore groups); resources that may be equally profitable for the puma, given its lower energetic needs associated with a smaller body size [13].

Additionally, the Expected Sociality axis revealed the most complex pattern. In addition to the centroid separation, the asymmetrical dispersion revealed significantly greater variability in puma populations. This functional plasticity, allowing pumas to more flexibly exploit niche refugia, functions as a compensatory equalizing mechanism that may reduce the fitness difference and promotes the persistence of the subordinate species. This mixed pattern of stabilization and equalization on some axes contrasts with Locomotor Habit and Weight Categories, which indicated a predominantly equalizing pattern, where predators are functionally similar with comparable population flexibility.

A second set of potent equalizing mechanisms was revealed by the specialization patterns. We found a strong synchrony in niche breadth for weight and sociality categories, suggesting that both predators respond similarly to environmental contexts, while acting independently for other traits. Crucially, this convergence does not escalate competition, which was demonstrated by the disconnection between niche breadth and overlap. This prevalence of neutral effects across most of the functional traits indicates that both predators can adjust their specialization level without a systematic increase or decrease in direct competition. This represents an important equalizing force, as it reduces average fitness differences [2]. The only exception to this pattern was a secondary stabilizing mechanism for Locomotor Habit, where jaguar generalization actively reduced overlap, reinforcing the mosaic of mechanisms within the functional axes of the trophic niche.

The functional prey group analysis indicates that taxonomic divergence (discussed earlier) is consistent with a primary stabilizing mechanism, permitting a high degree of functional convergence. This balance allows both predators to perform analogous ecological roles through different prey species, consistent with the discussion of shared functions within guilds and with the distinction between complementarity and apparent redundancy across multiple axes [62]. Convergence along the functional axis then is most parsimoniously interpreted as an equalizing mechanism, potentially reducing fitness disparities [2], even though we do not measure fitness differences directly. The puma trophic plasticity and the high functional overlap between jaguars and pumas are central to this effect. Together, these dynamics exemplify the niche complementarity hypothesis [6] and support the idea that the coexistence between potential competitors is sustained by a combined operation of multiple mechanisms that maintain species ecologically distinct yet sufficiently equivalent to persist [8].

### 4.3. From Coexistence Mechanisms to Conservation Actions

Our results highlight two key findings regarding the conservation and management of jaguars and pumas: (i) coexistence between jaguars and pumas promotes functional complementarity, supporting ecosystem stability and sustaining essential ecological processes; and (ii) effective conservation should safeguard both predators and their prey, as well as the ecological interactions linking them, to ensure the persistence of coexistence and overall ecosystem integrity.

Trophic segregation between jaguars and pumas, while driven by the reduction in direct competition, results in a broader and more complementary exploitation of the prey spectrum. This represents a clear example of functional complementarity, where partitioning enhances the range of ecological functions sustained by the predator guild [10]. Such complementarity not only has the potential to stabilize coexistence but also may amplify the scope and strength of top-down control over prey communities, demonstrating coexistence as a process that supports ecological resilience [11]. Therefore, joint preservation of apex predators generates cascading benefits such as mesopredator suppression and the promotion of functional diversity at lower trophic levels [17,64], thereby reinforcing ecological equilibrium.

Yet, despite this potential to jointly sustain key ecological functions, both species face vulnerabilities that threaten coexistence when external pressures exceed the self-regulating capacity of the system. Jaguars are classified as Near Threatened globally, persisting in approximately half of their historical range [20], while pumas are listed as Least Concern, with their original distribution comparatively better preserved [19]. Nevertheless, both species have undergone local population declines driven by habitat loss, depletion of native prey, and human–wildlife conflicts [19,20]. In many coexistence scenarios, populations are confined to fragmented and isolated habitats, where limitations of space and resources may intensify competitive interactions [11]. These vulnerabilities highlight that understanding the mechanisms sustaining coexistence is not only a theoretical challenge in trophic ecology but also a practical necessity for designing conservation strategies that ensure the persistence of both large felids.

Considering the ecological benefits of an intact predator guild, safeguarding the synergistic interaction between jaguars and pumas is as critical as protecting each species individually. Because maintaining equilibrium among potential competitors is fundamental, conservation and management actions disproportionately favor one predator, whether through its trophic base or other ecological aspects, may disrupt coexistence and compromise ecosystem integrity. This is evidenced by cascading consequences of predator loss and the resulting shifts competitive dynamics observed in other systems [10,11]. In this context, conservation approaches narrowly focused on single species may be ineffective, as they fail to capture the broader ecological interactions that sustain ecosystem balance [65] and, ultimately, the species themselves. The long-term persistence of both felids ultimately depends on conserving prey diversity at multiple scales, both to ensure the availability of energy-high prey that sustain jaguars and to maintain a broad spectrum of prey sizes and types consistent with the dietary plasticity of pumas. By safeguarding prey diversity across scales, conservation strategies can simultaneously meet the distinct trophic requirements of each predator and, in doing so, reinforce their coexistence. In line with our results, effective conservation must therefore aim to protect predators, prey communities, their habitats and the ecological interactions that connect them. The joint preservation of taxonomic and functional prey diversity is thus indispensable to ensure that jaguar–puma coexistence persists not only as redundant species but as functionally complementary predators that sustains the integrity of Neotropical ecosystems.

## 5. Conclusions

The coexistence between jaguars and pumas appears to be associated with a dynamic balance between trophic patterns consistent with stabilizing and equalizing mechanisms that operate across multiple ecological scales. Taxonomic segregation is most consistent with a primary stabilizing force, reducing direct competition through prey partitioning, while functional prey group convergence is most consistent with an equalizing pattern, potentially minimizing fitness differences and promoting persistence under competitive asymmetry. Together, these mechanisms create a multiscale equilibrium that sustains coexistence and reinforces the ecological roles of both large felids within Neotropical ecosystems.

From an applied perspective, the coexistence of these apex predators enhances functional complementarity within the predator guild, broadening the spectrum of ecological processes that maintain ecosystem resilience and stability. Therefore, conservation strategies should move beyond species-specific approaches and focus on preserving the diversity of prey communities and the trophic interactions that connect predators, prey and habitats. Safeguarding both taxonomic and functional prey diversity is essential to sustain the distinct trophic requirements of jaguars and pumas and to maintain the integrity of the ecosystems they regulate.

## Data Availability

The data supporting reported results are available in Tables S1 and S2.

## Supplementary Materials

The following supporting information can be downloaded at: https://www.mdpi.com/article/10.3390/biology15010031/s1, Table S1: List of studies containing diet data for sympatric populations of jaguar (*Panthera onca*) and puma (*Puma concolor*), included in the present study; Table S2: Taxonomic information, functional traits, and percentage of occurrence of each prey consumed by sympatric populations of jaguar (*Panthera onca*) and puma (*Puma concolor*), considered in the present study.

## Abbreviations

The following abbreviations are used in this manuscript:

NMDS	Non-metric Multidimensional Scaling
PERMANOVA	Permutational Multivariate Analysis of Variance
PERMDISP	Permutational Analysis of Multivariate Dispersion
